# On the association analysis of CNV data: a fast and robust family-based association method

**DOI:** 10.1186/s12859-017-1622-z

**Published:** 2017-04-18

**Authors:** Meiling Liu, Sanghoon Moon, Longfei Wang, Sulgi Kim, Yeon-Jung Kim, Mi Yeong Hwang, Young Jin Kim, Robert C. Elston, Bong-Jo Kim, Sungho Won

**Affiliations:** 1 Department of Applied Statistics, Chung-Ang University, Seoul, 156-756 South Korea; 2 Department of Bioinformatics and Computational Biology, Iowa State University, Ames, IA 50011 USA; 3 Division of Structural and Functional Genomics, Center for Genome Science, National Institute of Health, Cheongju-si, Chungcheongbuk-do 363-951 South Korea; 4 Interdisciplinary Program of Bioinformatics, Seoul National University, Seoul, 151-742 South Korea; 5 Naver Labs, 235 Pangyoyeok-ro, Bundang-gu, Seongnam-si, Gyeonggi-do, 13494 South Korea; 6 Department of Epidemiology and Biostatistics, Case Western Reserve University, Cleveland, OH 44106 USA; 7 Department of Public Health Science, Seoul National University, Seoul, 151-742 South Korea; 8 Institute of Health and Environment, Seoul National University, Seoul, 151-742 South Korea

**Keywords:** CNV, Association analysis, Score test, Hematocrit

## Abstract

**Background:**

Copy number variation (CNV) is known to play an important role in the genetics of complex diseases and several methods have been proposed to detect association of CNV with phenotypes of interest. Statistical methods for CNV association analysis can be categorized into two different strategies. First, the copy number is estimated by maximum likelihood and association of the expected copy number with the phenotype is tested. Second, the observed probe intensity measurements can be directly used to detect association of CNV with the phenotypes of interest.

**Results:**

For each strategy we provide a statistic that can be applied to extended families. The computational efficiency of the proposed methods enables genome-wide association analysis and we show with simulation studies that the proposed methods outperform other existing approaches. In particular, we found that the first strategy is always more efficient than the second strategy no matter whether copy numbers for each individual are well identified or not. With the proposed methods, we performed genome-wide CNV association analyses of hematological trait, hematocrit, on 521 Korean family samples.

**Conclusions:**

We found that statistical analysis with the expected copy number is more powerful than the statistic with the probe intensity measurements regardless of the accuracy of the estimation of copy numbers.

**Electronic supplementary material:**

The online version of this article (doi:10.1186/s12859-017-1622-z) contains supplementary material, which is available to authorized users.

## Background

Copy number variants (CNVs) are widely distributed throughout the human genome [1, 2] and have been considered as important genetic factors for human diseases [3, 4]. Several different methods, such as array comparative genomic hybridization (aCGH) and next generation sequencing, have been suggested to identify CNVs. Thanks to the recent improvement of sequencing technology, sequencing cost decreases very fast and becomes much cheaper. Furthermore, aCGH cannot detect aberrations such as mosaicism that do not result in copy number changes. However, in spite of this advantage of sequencing, aCGH is still cheaper and many array data have already been produced. Thus, it may be a cost effective choice at least for a while. In this report, we focus on CNV analysis with aCGH data–though the proposed method can be readily extended to other types of CNV data.

For aCGH data, gene copy numbers are not directly observed and have to be estimated with their intensity measures for association analyses. True unknown copy numbers will be called as unobserved copy numbers in the remainder of this report. CNV association requires estimation of copy numbers, and several algorithms, such as PennCNV [5], QuantiSNP [6], dChip [7] and GTC [8], have been developed to detect unobserved copy numbers. Then statistical methods such as linear regression and chi-square tests have been utilized to detect CNV association with estimated copy numbers [9]. Barns et al. [10] calculated the posterior probability for each possible copy number, and likelihoods weighted by these posterior probabilities were used to build a likelihood ratio test. As an alternative to CNV analysis using the estimated copy number, the probe intensity measurements can be used to detect the CNV association [11]. The probe intensity is assumed to be proportional to the unobserved copy number, and its distributions can be compared between affected and unaffected individuals. If copy numbers are correctly estimated, the analysis using the expected copy numbers seems to be an efficient choice. However, estimates of copy numbers are often uncertain and this effect has not been carefully considered in the statistical analysis [4]. In this report, we considered both approaches and compared them with simulation studies for a large variety of parameter settings.

For association analysis, phenotypic correlations between individuals have the effect of sample size reduction, and thus independent population-based samples have often been preferred to maximize the statistical efficiency. However, family-based association analyses have been useful for certain scenarios because family members are genetically homogeneous [12, 13]. For instance, FBAT-statistics based on the so-called within-family component [14] are robust in the presence of population substructure and they are often preferred, in particular for candidate gene studies. Within-family component indicates the distribution of non-founders’ genotype when their parental genotypes are conditioned. The distribution of founders’ genotype is called between-family component and the statistical power of FBAT-statistics has been improved by combining FBAT-statistics with the between-family component in a robust way [11, 15, 16]. This two-stage analysis can achieve efficiency comparable to that of independent samples. However, due to the assumption that between- and within- family components are equally informative, this method can suffer from statistical power of loss if the numbers of founders and nonfounders are different.

In this report we propose two statistics, *T*_1_ and *T*_2_, for CNV association analysis using family-based samples; for *T*_1_, the phenotypes are regressed on the expected copy number, and for *T*_2_ they are regressed directly on the probe intensity measurements. A random effect is included to model the phenotypic covariance between family members, and the variance components for the phenotype are estimated with a restricted likelihood. Our results show that statistical analysis with the expected copy number is usually more efficient than the statistic with probe intensity measurements. We applied the proposed methods to detect CNV association with a hematology-related trait, hematocrit, in Korean family-based samples.

## Methods

### Notations and the disease model

We assume that *K* intensity measurements are observed at a particular CNV region for each individual, there are *n* families, and *n*_*i*_ individuals in family *i*. For simplicity, we consider only trio families, but the methods can be extended to large extended families. We assume that *j* = 1, 2 indicates the parents in each family. We let *x*_*ijk*_ indicate the observed intensity measurement on probe *k* for individual *j* in family *i*. **X**_*ij*_ indicates the column vector, (*x*_*ij*1_,…,*x*_*ijK*_)^*T*^, for individual *j* in family *i*. We let *λ*_*ij*_ be the unobserved copy number for individual *j* in family *i*, and denote a set of possible realizations of *λ*_*ij*_ and their corresponding frequencies respectively as ***C*** and Θ. We denote the phenotype for individual *j* in family *i* by *y*_*ij*_, and let **Z**_*ij*_ be a vector of measured environmental factors, including an intercept as the first element. The intensity matrix, **X**_*i*_, and phenotype vector, **Y**_*i*_, for family *i* are respectively defined as ${\mathbf{X}}_i={\left({\mathbf{X}}_{i1}^T,\dots, {\mathbf{X}}_{i{ n}_i}^T\right)}^T$ and ${\mathbf{Y}}_i={\left({y}_{i1},\dots, {y}_{i{ n}_i}\right)}^T$. We include a random effect, **b**_*i*_, to allow for the phenotypic correlation between family members. **λ**_*i*_ and **ε**_*i*_ indicate respectively an unobserved copy number vector and a measurement error vector for family members in family *i*. If we let *N* = Σ_*i*_ *n*_*i*_, an *N* × *K* design matrix **X** and an *N* × 1 vector **Y** are respectively obtained by stacking all **X**_*i*_ and **Y**_*i*_ vertically. **λ**, **b** and **ε** are *N* × 1 vectors and are obtained by stacking all **λ**_*i*_, **b**_*i*_ and **ε**_*i*_ vertically.

#### Signal model

We assumed that there are some correlations among the probe intensity measurements and the correlation matrix is assumed to be unstructured. We let ${\boldsymbol{\upgamma}}_{\lambda_{ij}}$ and ${\boldsymbol{\Sigma}}_{\lambda_{ij}}$ be a *K* × 1 mean vector and a *K* × *K* variance-covariance matrix of the intensity measurements. We assume that **X**_*ij*_|*λ*_*ij*_ are identical and independently distributed for *i* and *j*, and

$$ {\mathbf{X}}_{ij}\Big|{\lambda}_{ij}\sim \mathcal{N}\left({\boldsymbol{\upgamma}}_{ij},{\boldsymbol{\Sigma}}_{ij}\right). $$

If we assume that the correlation matrix is **R**, the variance-covariance matrix can be expressed as ${\boldsymbol{\Sigma}}_{\lambda_{ij}}={\mathbf{D}}_{\lambda_{ij}}\mathbf{R}{\mathbf{D}}_{\lambda_{ij}}$, where

$$ {\mathbf{D}}_{\lambda_{ij}}=\left[\begin{array}{ccc}\hfill {\sigma}_{1{\lambda}_{ij}}\hfill & \hfill 0\hfill & \hfill \cdots \hfill \\ {}\hfill 0\hfill & \hfill {\sigma}_{2{\lambda}_{ij}}\hfill & \hfill \mathbf{O} \hfill \\ {}\hfill \vdots \hfill & \hfill \mathbf{O} \hfill & \hfill \mathbf{O} \hfill \end{array}\right]. $$

The parameters for ${\boldsymbol{\Sigma}}_{\lambda_{ij}}$ will be denoted by **Σ**, and this proposed model will be called the signal model in the remainder of this manuscript.

#### Phenotype model

We assume that phenotypes are quantitative. We consider a standard linear mixed model for phenotypes that consists of CNV effects, additive polygenic effects, and measurement error. If we denote the *w* × *w* identity matrix by **I**_*w*_, the measurement error **ε** is assumed to follow the multivariate normal distribution with mean **0** and variance *σ*_*ε*_^2^ **I**_*N*_. The phenotypic correlations between family members are usually explained by a polygenic effect, **b**, and we assume **b** follows a multivariate normal distribution. We let ${\pi}_{ij{ j}^{\prime }}$ be the kinship coefficient between individuals *j* and *j*′ in family *i*, we let *d*_*ij*_ be the inbreeding coefficient for individual *j* in family *i*, we denote **Ф**_*i*_ by the matrix

$$ \left[\begin{array}{cccc}\hfill 1+{d}_{i1}\hfill & \hfill 2{\pi}_{i12}\hfill & \hfill \cdots \hfill & \hfill 2{\pi}_{i1{n}_i}\hfill \\ {}\hfill 2{\pi}_{i21}\hfill & \hfill 1+{d}_{i2}\hfill & \hfill \cdots \hfill & \hfill 2{\pi}_{i2{n}_i}\hfill \\ {}\hfill \vdots \hfill & \hfill \vdots \hfill & \hfill \ddots \hfill & \hfill \vdots \hfill \\ {}\hfill 2{\pi}_{i{ n}_i1}\hfill & \hfill 2{\pi}_{i{ n}_i2}\hfill & \hfill \cdots \hfill & \hfill 1+{d}_{i{ n}_i}\hfill \end{array}\right], $$

and we let

$$ \boldsymbol{\Phi} =\left[\begin{array}{cccc}\hfill {\boldsymbol{\Phi}}_1\hfill & \hfill 0\hfill & \hfill 0\hfill & \hfill \cdots \hfill \\ {}\hfill 0\hfill & \hfill {\boldsymbol{\Phi}}_2\hfill & \hfill 0\hfill & \hfill \cdots \hfill \\ {}\hfill 0\hfill & \hfill 0\hfill & \hfill {\boldsymbol{\Phi}}_3\hfill & \hfill \cdots\ \hfill \\ {}\hfill \vdots \hfill & \hfill \vdots \hfill & \hfill \vdots \hfill & \hfill \mathrm{O}\hfill \end{array}\right]. $$

The kinship coefficient between two subjects indicates the probability that two alleles randomly selected from each subject are identical by decent, and the inbreeding coefficient of a subject means the probability that his or her two alleles are identical by descent. Then, if we let the variance of the polygenic effect be *σ*_*g*_^2^, **b** follows the multivariate normal distribution with mean **0** and variance covariance matrix, *σ*_*g*_^2^ **Ф**. In the presence of population substructure, the empirical correlation matrix estimated with large-scale SNP data can replace **Ф** to provide robustness to the proposed method [17, 18]. If we condition on the true copy number vector **λ**, the linear model for the phenotype is

1 $$ \mathbf{Y}=\mathbf{Z}\boldsymbol{\upalpha } +\boldsymbol{\uplambda} \beta +\mathbf{b}+\boldsymbol{\upvarepsilon},\ \mathrm{where}\ \mathbf{b}\sim \mathcal{N}\left(0,{\sigma}_g^2\boldsymbol{\Phi} \right),\boldsymbol{\upvarepsilon} \sim \mathcal{N}\left(0,{\sigma}_{\varepsilon}^2{\mathbf{I}}_N\right). $$

#### Copy number model

For disease copy number region we assume that there are *M* different unobserved copy numbers in the population. We further assume that the frequency of subjects with *c*_*m*_ copy numbers is *θ*_*m*_ in the population. We let ***C*** = {*c*_1_, …, *c*_*M*_} and Θ = {*θ*_1_, …, *θ*_*M*_}, where *θ*_1_ + … + *θ*_*M*_ = 1. We denote maternal and paternal copy numbers of individual *j* in family *i* by *λ*_*ij*_^1^ and *λ*_*ij*_^2^ respectively, and we assume that *λ*_*ij*_ (= *λ*_*ij*_^1^ + *λ*_*ij*_^2^) for founders follows the multinomial distribution under Hardy-Weinberg equilibrium. It should be noted that *λ*_*ij*_ can be any element in ***C***. We assume no *de novo* CNVs and we assume that parental CNVs are transmitted to their offspring in a Mendelian fashion. For simplification, we consider nuclear families but the proposed method can be easily extended to the extended families. The probability of the ordered copy numbers for subjects in nuclear family *i* becomes

$$ \begin{array}{c}\hfill P\left(\left({\lambda}_{i1}^1,{\lambda}_{i1}^2\right),\left({\lambda}_{i2}^1,{\lambda}_{i2}^2\right),\dots, \left({\lambda}_{i{ n}_i}^1,{\lambda}_{i{ n}_i}^2\right)\right)= P\left(\left({\lambda}_{i1}^1,{\lambda}_{i1}^2\right)\right) P\left(\left({\lambda}_{i2}^1,{\lambda}_{i2}^2\right)\right)\hfill \\ {}\hfill \times {\displaystyle \prod_{j=1}^{n_i}} P\left(\left({\lambda}_{i j}^1,{\lambda}_{i j}^2\right)\Big|\left({\lambda}_{i1}^1,{\lambda}_{i1}^2\right),\left({\lambda}_{i2}^1,{\lambda}_{i2}^2\right)\right).\hfill \end{array} $$

Here, for *j* = 1 or 2,

$$ P\left(\left({\lambda}_{ij}^1,{\lambda}_{ij}^2\right)\right)=\left\{\begin{array}{cc}\hfill {\theta}_m^2,\hfill & \hfill if\ {\lambda}_{ij}^1={\lambda}_{ij}^2={c}_m\hfill \\ {}\hfill 2{\theta}_m{\theta}_{m^{\prime }},\hfill & \hfill if\ {\lambda}_{ij}^1={c}_m,{\lambda}_{ij}^2={c}_{m^{\prime }}\hfill \\ {}\hfill \hfill & \hfill {c}_m\ne {c}_{m^{\prime }}\hfill \end{array}\right., $$

and for *j* = 3, …, *n*_*i*_,

$$ P\left(\left({\lambda}_{i j}^1,{\lambda}_{i j}^2\right)\Big|\left({\lambda}_{i1}^1,{\lambda}_{i1}^2\right),\left({\lambda}_{i2}^1,{\lambda}_{i2}^2\right)\right)=\left\{\begin{array}{cc}\hfill 1/4,\hfill & \hfill if\ {\lambda}_{i j}^1={\lambda}_{i1}^l,{\lambda}_{i j}^2={\lambda}_{i2}^{l^{\prime }},\hfill \\ {}\hfill \hfill & \hfill l=1,2,{l}^{\prime }=1,2\hfill \\ {}\hfill 0,\hfill & \hfill otherwise\hfill \end{array}\right.. $$

We let Λ_*ij*_ be the set of possible maternal and paternal copy number pairs for individual *j* in family *i*, for which the sum is equal to *λ*_*ij*_ *,* as follows:

$$ {\Lambda}_{ij}=\left\{\left({\lambda}_{ij}^{*1},{\lambda}_{ij}^{*2}\right)\Big|{\lambda}_{ij}^{*1}+{\lambda}_{ij}^{*2}={\lambda}_{\mathrm{ij}}\right\}. $$

Then the joint probability of ${\lambda}_{i1},\dots, {\lambda}_{i{ n}_i}$ for individuals in family *i* is

$$ P\left({\lambda}_{i1},\dots, {\lambda}_{i{ n}_i}\right)={\displaystyle \sum_{\left({\lambda}_{i1}^{*1},{\lambda}_{i1}^{*2}\right)\in {\Lambda}_{i1}}}\cdots {\displaystyle \sum_{\left({\lambda}_{i{ n}_i}^{*1},{\lambda}_{i{ n}_i}^{*2}\right)\in {\Lambda}_{i{ n}_i}}} P\left(\left({\lambda}_{i1}^{*1},{\lambda}_{i1}^{*2}\right),\dots, \left({\lambda}_{i{ n}_i}^{*1},{\lambda}_{i{ n}_i}^{*2}\right)\right). $$

If we assume that **λ** and **b** are missing values for the EM algorithm, our full likelihood is

2 $$ \begin{array}{c}\hfill f\left(\mathbf{X},\mathbf{Y},\boldsymbol{\uplambda}, \mathbf{b}\Big|\mathbf{Z},\boldsymbol{\Phi}, \boldsymbol{\upalpha}, \beta, \boldsymbol{\upgamma}, \boldsymbol{\Sigma}, \boldsymbol{\Theta} \right)\hfill \\ {}\hfill = f\left(\mathbf{X}\Big|\boldsymbol{\uplambda}; \boldsymbol{\upgamma}, \boldsymbol{\Sigma} \right)\cdot f\left(\mathbf{Y},\mathbf{b}\Big|\mathbf{Z},\boldsymbol{\uplambda}; \boldsymbol{\upalpha}, \beta, {\sigma}_g^2,{\sigma}_{\varepsilon}^2\right)\cdot f\left(\boldsymbol{\uplambda} \Big|\boldsymbol{\Theta} \right)\hfill \end{array}. $$

### Parameter estimation with the EM algorithm

To derive a score test for CNV association analysis, *β* in the phenotype model was assumed to be 0, and the variance component parameters were estimated with the restricted maximum likelihood (REML) method. The copy number vector **λ** and the random effect vector **b** are considered as missing variables for the EM algorithm, and the conditional expectation of a complete data log-likelihood was maximized to estimate all the parameters. Individuals were separated with K-means clustering [19], and the empirical mean and co-variance matrix were used as the initial values for the signal model.

In the expectation step, we calculate posterior probabilities for each possible value of the unobserved copy number using the estimates from the previous iteration. We use the superscript (*ω*) to indicate the estimate at the *ω-*th iteration. The posterior probability of **λ** is obtained by

$$ \begin{array}{l} P\left(\boldsymbol{\uplambda} \Big|\mathbf{X},\mathbf{Y},\mathbf{Z},\boldsymbol{\Phi}, {\boldsymbol{\upalpha}}^{\left(\omega \right)},{\widehat{\beta}}^{\left(\omega \right)},{\widehat{\boldsymbol{\upgamma}}}^{\left(\omega \right)},{\widehat{\boldsymbol{\Sigma}}}^{\left(\omega \right)},{\widehat{\boldsymbol{\Theta}}}^{\left(\omega \right)}\right)\hfill \\ {}=\frac{f\left(\mathbf{X},\mathbf{Y},\boldsymbol{\uplambda} \Big|\mathbf{Z},\boldsymbol{\Phi}, {\widehat{\boldsymbol{\upalpha}}}^{\left(\omega \right)},{\widehat{\beta}}^{\left(\omega \right)},{\widehat{\boldsymbol{\upgamma}}}^{\left(\omega \right)},{\widehat{\boldsymbol{\Sigma}}}^{\left(\omega \right)},{\widehat{\boldsymbol{\Theta}}}^{\left(\omega \right)}\right)}{{\displaystyle {\sum}_{\lambda^{\prime }}} f\left(\mathbf{X},\mathbf{Y},{\boldsymbol{\uplambda}}^{\mathbf{\prime}}\Big|\mathbf{Z},\boldsymbol{\Phi}, {\widehat{\boldsymbol{\upalpha}}}^{\left(\omega \right)},{\widehat{\beta}}^{\left(\omega \right)},{\widehat{\boldsymbol{\upgamma}}}^{\left(\omega \right)},{\widehat{\boldsymbol{\Sigma}}}^{\left(\omega \right)},{\widehat{\boldsymbol{\Theta}}}^{\left(\omega \right)}\right)}.\hfill \end{array} $$

Under the null hypothesis, this posterior probability becomes

$$ \begin{array}{l} P\left(\boldsymbol{\uplambda} \Big|\mathbf{X},\mathbf{Y},\mathbf{Z},\boldsymbol{\Phi}, {\widehat{\boldsymbol{\upalpha}}}^{\left(\omega \right)},\beta =0,{\widehat{\boldsymbol{\upgamma}}}^{\left(\omega \right)},{\widehat{\boldsymbol{\Sigma}}}^{\left(\omega \right)},{\widehat{\boldsymbol{\Theta}}}^{\left(\omega \right)}\right)\hfill \\ {}=\frac{f\left(\mathbf{X}\Big|\boldsymbol{\uplambda}; {\widehat{\boldsymbol{\upgamma}}}^{\left(\omega \right)},{\widehat{\boldsymbol{\Sigma}}}^{\left(\omega \right)}\right) f\left(\boldsymbol{\uplambda} \Big|{\widehat{\boldsymbol{\Theta}}}^{\left(\omega \right)}\right)}{{{\displaystyle {\sum}_{\lambda}}}^{\prime } f\left(\mathbf{X}\Big|{\boldsymbol{\uplambda}}^{\mathbf{\prime}};{\widehat{\boldsymbol{\upgamma}}}^{\left(\omega \right)},{\widehat{\boldsymbol{\Sigma}}}^{\left(\omega \right)}\right) f\left({\boldsymbol{\uplambda}}^{\mathbf{\prime}}\Big|{\widehat{\boldsymbol{\Theta}}}^{\left(\omega \right)}\right)}\ .\hfill \end{array} $$

The copy number with the largest posterior density was assumed to be the true copy number ${\widehat{\boldsymbol{\uplambda}}}^{\left(\omega +1\right)}$ for each individual in the (*ω* + 1)*-*th iteration. For the missing variable **b**, if we let **V** = *σ*_*g*_^2^ **Φ** + *σ*_*ε*_^2^ **I**_*N*_ and **e** = **Y** − **Zα** − **λ** *β*, the posterior mean of **b** in the (*ω* + 1)*-*th iteration is estimated as

$$ {\widehat{\mathbf{b}}}^{\left(\omega +1\right)}={\widehat{\sigma}}_g^{\left(\omega \right)2}\boldsymbol{\Phi} {\widehat{\mathbf{V}}}^{\left(\omega \right)-1}{\widehat{\mathbf{e}}}^{\left(\omega \right)}. $$

In the maximization step, all parameters are estimated by maximizing the expected log-likelihood of

$$ f\left(\mathbf{X},\mathbf{Y},{\boldsymbol{\uplambda}}^{\left(\omega \right)},{\mathbf{b}}^{\left(\omega \right)}\Big|\mathbf{Z},\boldsymbol{\Phi}, {\widehat{\boldsymbol{\upalpha}}}^{\left(\omega \right)},{\widehat{\beta}}^{\left(\omega \right)},{\widehat{\boldsymbol{\upgamma}}}^{\left(\omega \right)},{\widehat{\boldsymbol{\Sigma}}}^{\left(\omega \right)},{\widehat{\boldsymbol{\Theta}}}^{\left(\omega \right)}\right). $$

**γ** and **Σ** are updated by the sample mean and sample variance-covariance matrix. **α** and *β* in the phenotype model are estimated by

$$ \left({\widehat{\boldsymbol{\upalpha}}}^{\left(\omega +1\right)},{\widehat{\beta}}^{\left(\omega +1\right)}\right)={\left[\left[\begin{array}{c}\hfill Z\hfill \\ {}\hfill {\widehat{\boldsymbol{\uplambda}}}^{\left(\omega \right)}\hfill \end{array}\right]{\widehat{\mathbf{V}}}^{\left(\omega \right)-1}\left(\begin{array}{cc}\hfill \mathbf{Z}\hfill & \hfill {\widehat{\boldsymbol{\uplambda}}}^{\left(\omega \right)}\hfill \end{array}\right)\right]}^{-1}\left[\begin{array}{c}\hfill Z\hfill \\ {}\hfill {\widehat{\boldsymbol{\uplambda}}}^{\left(\omega \right)}\hfill \end{array}\right]{\widehat{\mathbf{V}}}^{\left(\omega \right)-1}\mathbf{Y}. $$

The variance parameters, *σ*_*g*_^2^ and *σ*_*ε*_^2^, are updated as

$$ \begin{array}{c}\hfill {\widehat{\sigma}}_g^{\left(\omega +1\right)2}={\widehat{\sigma}}_g^{\left(\omega \right)2}+\frac{1}{N}\mathrm{tr}\left({\widehat{\mathbf{b}}}^{\left(\omega \right)}{\widehat{\mathbf{b}}}^{\left(\omega \right) T}-{\widehat{\sigma}}_g^{\left(\omega \right)4}\boldsymbol{\Phi} {\widehat{\mathbf{P}}}^{\left(\omega \right)}\boldsymbol{\Phi} \right)\hfill \\ {}\hfill {\widehat{\sigma}}_{\varepsilon}^{\left(\omega +1\right)2}={\widehat{\sigma}}_{\varepsilon}^{\left(\omega \right)2}+\frac{1}{N}\mathrm{tr}\left({\widehat{\mathbf{e}}}^{\left(\omega \right)}{\widehat{\mathbf{e}}}^{\left(\omega \right) T}-{\widehat{\sigma}}_{\varepsilon}^{\left(\omega \right)4}{\widehat{\mathbf{P}}}^{\left(\omega \right)}\right),\hfill \end{array} $$

where ${\widehat{\mathbf{P}}}^{\left(\omega \right)}={\widehat{\mathbf{V}}}^{\left(\omega -1\right)-1}-{\widehat{\mathbf{V}}}^{\left(\omega -1\right)-1}\mathbf{X}{\left({\mathbf{X}}^T{\widehat{\mathbf{V}}}^{\left(\omega -1\right)-1}\mathbf{X}\right)}^{-1}{\mathbf{X}}^T{\widehat{\mathbf{V}}}^{\left(\omega -1\right)-1}$. Last, *θ*_*k*_ is updated with the following best linear unbiased estimator [20]:

$$ \begin{array}{c}\hfill {\widehat{\theta}}_k^{\left(\omega +1\right)}=\frac{1}{2}{\left({1}_N^T{\boldsymbol{\Phi}}^{-1}{1}_N\right)}^{-1}{1}_N^T{\boldsymbol{\Phi}}^{-1}\hfill \\ {}\hfill \times \left[\begin{array}{c}\hfill P\left({\lambda}_{11}^1={c}_k\Big|\mathbf{X},\mathbf{Y},\mathbf{Z},\boldsymbol{\Phi}, {\widehat{\boldsymbol{\upalpha}}}^{\left(\omega \right)},{\widehat{\beta}}^{\left(\omega \right)},{\widehat{\boldsymbol{\upgamma}}}^{\left(\omega \right)},{\widehat{\boldsymbol{\Sigma}}}^{\left(\omega \right)},{\widehat{\boldsymbol{\Theta}}}^{\left(\omega \right)}\right)\hfill \\ {}\hfill \vdots \hfill \\ {}\hfill P\left({\lambda}_{n{ n}_n}^1={c}_k\Big|\mathbf{X},\mathbf{Y},\mathbf{Z},\boldsymbol{\Phi}, {\widehat{\boldsymbol{\upalpha}}}^{\left(\omega \right)},{\widehat{\beta}}^{\left(\omega \right)},{\widehat{\boldsymbol{\upgamma}}}^{\left(\omega \right)},{\widehat{\boldsymbol{\Sigma}}}^{\left(\omega \right)},{\widehat{\boldsymbol{\Theta}}}^{\left(\omega \right)}\right)\hfill \end{array}\right].\hfill \end{array} $$

### Identifying the number of clusters

The optimal *M* was chosen with the silhouette score which quantifies whether objects in the same cluster stay together and objects in different clusters are well separated [21]. We denote the Euclidean distance between **X**_*ij*_ and ${\mathbf{X}}_{i^{\prime }{j}^{\prime }}$ by *d*_*ij*,*i* ' *j* '_, and denote the number of individuals whose copy numbers are *c*_*m*_ by *n*(*c*_*m*_). If the estimated copy number ${\widehat{\lambda}}_{ij}$ for individual *j* in family *i* is assumed to be *c*_*m*_, we let the average distance to the rest of the cluster be

$$ {a}_{i j}=\frac{1}{n\left({c}_m\right)}{\displaystyle \sum_{\left\{\left({i}^{\prime },{j}^{\prime}\right)\Big|{\widehat{\lambda}}_{i^{\prime }{j}^{\prime }}={c}_m\right\}}}{d}_{i j,{i}^{\prime }{j}^{\prime }}, $$

and the minimum average distance to other clusters be

$$ {b}_{i j}= \min \left\{\left.\frac{{\displaystyle {\sum}_{\left\{\left({i}^{\prime },{j}^{\prime}\right)\Big|{\widehat{\lambda}}_{i^{\prime }{j}^{\prime }}={c}_m\right\}}}{d}_{i j,{i}^{\prime }{j}^{\prime }}}{n\left({c}_{m^{\prime }}\right)}\right|{m}^{\prime}\ne m,\ {m}^{\prime }=1,\dots, M\right\}. $$

Then the silhouette score for individual *j* in family *i* is defined as

$$ s i{l}_{ij}=\frac{b_{ij}-{a}_{ij}}{ \max \left\{{a}_{ij},{b}_{ij}\right\}}. $$

If *sil*_*ij*_ is close to one, it indicates that the corresponding individual is well-clustered, whereas if *sil*_*ij*_ is close to −1, it means that the individual is badly clustered. If *sil*_*ij*_ is close to zero, there may exist a better cluster for the corresponding individual. Therefore, we first estimated the copy numbers for each individual for different choices of *M*. Then we calculated silhouette scores for all the individuals, and the value of *M* that maximized the mean silhouette score was considered as the optimal choice.

### Statistical inference

The Wald and likelihood ratio tests for the proposed likelihood are computationally intensive, and CNV association analysis with large families may not be feasible on a genome-wide scale. Therefore, we provide two score statistics based on Eq. (2); one is based on the estimated copy number and the other is based on the probe intensity measurement itself. First, the copy numbers and parameters for variance components are estimated from the likelihood under the null hypothesis. The expected copy numbers are assumed to be the unknown true copy numbers. Then Rao’s score test statistic is

$$ {T}_1={\left({\left(\mathbf{Y}-\mathbf{Z}\boldsymbol{\upalpha } \right)}^T{\mathbf{V}}^{-1}\boldsymbol{\uplambda} \right)}^T{\left({\boldsymbol{\uplambda}}^T{\mathbf{V}}^{-1}\boldsymbol{\uplambda} -{\left({\mathbf{Z}}^T{\mathbf{V}}^{-1}\boldsymbol{\uplambda} \right)}^T{\left({\mathbf{Z}}^T{\mathbf{V}}^{-1}\mathbf{Z}\right)}^{-1}\left({\mathbf{Z}}^T{\mathbf{V}}^{-1}\boldsymbol{\uplambda} \right)\right)}^{-1}\left({\left(\mathbf{Y}-\mathbf{Z}\boldsymbol{\upalpha } \right)}^T{\mathbf{V}}^{-1}\boldsymbol{\uplambda} \right), $$

and *T*_1_ follows the chi-square distribution with a single degree of freedom under *H*_0_ (See Additional file 1: Text 1 for details). If there exists no inverse matrix of **V**, the generalized inverse matrix [22] can be utilized.

However, *T*_1_ is based on the estimates of the expected copy numbers and its performance may depend on the accuracy of $\widehat{\boldsymbol{\uplambda}}$. We therefore also provide the statistic *T*_2_, based directly on the probe intensity measurements. It should be noted that, contrary to *T*_1_, *T*_2_ does not need one to estimate the unknown copy number and the computation is less intensive. We let **Ψ** be the empirical variance-covariance matrix between individuals and **I**_*N*_ be the *N × N* dimensional identical matrix,

$$ \begin{array}{c}\hfill \mathbf{v}=\frac{\mathrm{tr}\left(\left({\mathbf{I}}_N-{\mathbf{S}}_2\right)\boldsymbol{\Psi} {\left({\mathbf{I}}_N-{\mathbf{S}}_2\right)}^T{\mathbf{V}}^{-1}\left({\mathbf{I}}_N-{\mathbf{S}}_1\right)\right)}{\mathrm{tr}\left(\left({\mathbf{I}}_N-{\mathbf{S}}_2\right)\boldsymbol{\Psi} {\boldsymbol{\Phi}}^{-1}\right)}\hfill \\ {}\hfill \times {\mathbf{X}}^T{\left({\mathbf{I}}_N-{\mathbf{S}}_2\right)}^T{\boldsymbol{\Phi}}^{-1}\left({\mathbf{I}}_N-{\mathbf{S}}_2\right)\mathbf{X},\hfill \end{array} $$

and

$$ {\mathbf{u}}^T={\mathbf{Y}}^T{\left({\mathbf{I}}_N-{\mathbf{S}}_1\right)}^T{\mathbf{V}}^{-1}\left({\mathbf{I}}_N-{\mathbf{S}}_2\right)\mathbf{X}, $$

where

$$ {\mathbf{S}}_1=\mathbf{Z}{\left({\mathbf{Z}}^T{\mathbf{V}}^{-1}\mathbf{Z}\right)}^{-1}{\mathbf{Z}}^T{\mathbf{V}}^{-1},\ \mathrm{and}\ {\mathbf{S}}_2={1}_N{\left({1}_N^T{\boldsymbol{\Phi}}^{-1}{1}_N\right)}^{-1}{1}_N^T{\boldsymbol{\Phi}}^{-1}. $$

If we denote the rank of **v** by *r*, *T*_2_ is defined by

$$ {T}_2={\mathbf{u}}^T{\mathbf{v}}^{-1}\mathbf{u}\sim {\chi}^2\left( df= r\right)\ \mathrm{under}\ {H}_0. $$

The detailed derivation of *T*_2_ is shown in Additional file 1: Text 2. In particular, we can utilize a transformed value for **X** in *T*_2_. For instance, the mean intensity measurement over all probes or the first principal component (PC) score can be utilized, and then *T*_2_ follows the chi-square distributions with a single degree of freedom. Implementation of the methods is assembled in an R package PedCNV, which is available from CRAN.

### Simulation studies

#### Data generation

We conducted simulation studies to evaluate the performance of the proposed methods and, for computational simplicity, we simulated just 300 parent-offspring trios. We considered two scenarios; (1) *M* = 3, ***C*** = {0, 1, 2} and Θ = {(1 − *θ*)^2^, 2*θ*(1 − *θ*), *θ* ^2^}, and (2) *M* = 6, ***C*** = {0, 1, 2, 3, 4, 5} and Θ = {(1 − *θ*)^5^, 5*θ*(1 − *θ*)^4^, 10*θ* ^2^(1 − *θ*)^3^, 10*θ* ^3^(1 − *θ*)^2^, 5*θ* ^4^(1 − *θ*), *θ* ^5^}. Copy numbers for offspring were generated with simulated Mendelian transmission. We assumed *K* = 7 probe intensities were measured for a CNV region, and each intensity, *x*_*ijk*_, was generated from a normal distribution with

$$ E\left({x}_{ij k}\right)=\left\{\begin{array}{ll}{s}_k{\lambda}_{ij}+{p}_{\lambda_{ij}},\hfill & k=1,2,3\hfill \\ {}{s}_k+{p}_{\lambda_{ij}},\hfill & k=4,5,6,7\hfill \end{array}\right.,\mathrm{v}\mathrm{a}\mathrm{r}\left({x}_{ij k}\right)={\left( z\cdot {\lambda}_{ij}+{q}_v\right)}^2. $$

Here the dissimilarity between probe intensity measurements in different clusters for probe *k* is proportional to the value of *s*_*k*_ and we considered three scenarios by using three different choices of *s*_*k*_: badly separated clusters (BSC), moderately separated clusters (MSC) and well separated clusters (WSC). The different means for the probe intensities were provided by ${p}_{\lambda_{ij}}$ generated from *N*(0, 0.9(*λ*_*ij*_ + 1.5)^2^). The variance of each probe intensity measurement was provided by *q*_*v*_ generated from Γ(0.025,0.0016^2^). The parameter settings in the signal model are described in Additional file 1: Table S1.

Phenotypes were generated based on Eq. (1). For the phenotype model, we assumed that there was a single covariate for **Z** which was independently generated for each individual from the standard normal distribution. *σ*_*ε*_^2^ and *σ*_*g*_^2^ were assumed to be 1. For our simulations, we considered trios and **Φ**_*i*_ becomes

$$ \left[\begin{array}{ccc}\hfill 1\hfill & \hfill 0\hfill & \hfill .5\hfill \\ {}\hfill 0\hfill & \hfill 1\hfill & \hfill .5\hfill \\ {}\hfill .5\hfill & \hfill .5\hfill & \hfill 1\hfill \end{array}\right]. $$

### Analysis of a hematological trait

#### Subjects

Hematocrit indicates the volume percentage of red blood cells in blood and red blood cells transfer oxygen from the lungs to body tissues. Some diseases such as anemia are related to hematocrit and we conducted association analyses of hematocrit to identify CNVs related to anemia. We used the same DNA samples as were used in Lee et al. [23]. Five hundred fifty-one individuals from 59 families including 216 Granular corneal dystrophy type 2 patients and 324 unaffected controls were genotyped with Illumina HumanCNV 370 K-Duo Beadchip. Clinical information for 30 individuals was missing. Therefore, 521 individuals were used for the association analysis. All subjects enrolled in this study were of Korean ethnicity. Basic characteristics of our samples are summarized in Table 1.

**Table 1:** Basic characteristics of study participants and hema-tological trait

Variables	Discovery (family)	Replication (cohort)
Sample size (n)	521	4694
Age (years)	38.2 ± 18.3	54.0 ± 9.0
Male (%)	45.7%	47.1%
Hematocrit (%)	41.3 ± 4.3	41.1 ± 4.5

#### CNV discovery

All samples were genotyped with NimbleGen HD2 3 × 720 K aCGH which contains more than 720,000 probes. Around 360,000 probes were designed based on previously reported CNVs, and the other probes were spaced uniformly throughout the whole genome as a backbone. Sample NA10851obtained from the HapMap lymphoblastoid cell line (LCL) DNA was used as a reference, and NimbleScan version 2.5 was used to process the array image files (.tif) according to the manufacturer’s protocol. Extracted signal intensity was transformed to log2 ratio with hg18/NCBI build 36. Subsequently, we set the log2 ratio thresholds less than −0.25 for a deletion and greater than 0.25 for a duplication, with more than 10 consecutive probes required to assign a CNV.

#### CNV selection

We used a reciprocal overlap threshold > 50% to find CNVs with similar boundaries for association analysis. According to this threshold, clusters of overlapping CNVs at the sample level are merged into one CNV. Overlapping CNVs with very different sizes and sequentially connected CNVs were excluded from further study. Moreover, we selected CNV clusters which are well-separated and have multi-class CNVs in order to assign individuals to copy-number classes with high confidence [24]. In total 500 CNVs were utilized for association analyses.

#### CNV association

PedCNV was applied to an association study with a hematological trait: hematocrit (Hct). The association of CNVs with Hct was analyzed using *T*_1_ and *T*_2_, with age, age^2^ and sex included as covariates. The resulting statistics were adjusted by using genomic control to allow for population substructure.

#### CNV validation by PCR experiment

To confirm CNV genotypes, a PCR using the AccuPrime Taq DNA Polymerase High Fidelity (invitrogen, CA, USA) was performed on 10–16 individuals selected from each cluster (Additional file 1: Figure S1 (A)). The primers were designed to give rise to amplicons with different lengths to detect both the deleted (690 bp) and normal (1519 bp) alleles (Additional file 1: Table S2). Genomic locations for designed primers based on human genome assembly hg18 were converted to those based on hg19 by liftOver of the UCSC genome browser. PCR was carried out on a GeneAmp PCR system 9700 (Applied Biosystems, Calif., USA) with the following PCR conditions: 5 min at 95 °C, followed by 33 cycles of 30s at 95 °C, 30s at 60 °C, 2 min at 68 °C, and final extension at 68 °C for 7 min. The resulting PCR products were visualized by electrophoresis separation on a 1.5% agarose gel with Safe-Pinky DNA gel staining solution (Genedepot, TX, USA). Moreover, to confirm exact break-points of the CNVs, PCR products were sequenced using an ABI 3730 DNA analyzer (Applied Biosystems, CA, USA).

#### Replication study

We have previously implemented KGVDB, which includes 3601 multi-class CNVs and their tagging SNPs, from 4694 community-based cohort samples, as a part of the Korean Genome Epidemiology Study (KoGES) [25]. We used these unrelated individual samples to pursue replication of the identified CNV from the discovery association study. Table 1 shows a summary of the participants’ characteristics. In short, all the 4694 samples were also genotyped with NimbleGen HD2 3 × 720 K aCGH. The NA10851 sample was again used as a reference. NimbleScan version 2.5 was used to extract signal intensity. Subsequently, quality control, such as normalization and waviness correction, was conducted using the R package (http://cran.r-project.org) and WaveNorm [26]. For CNV detection, the Genome Alteration Detection Analysis algorithm (GADA) was used with T = 10, alpha = 0.2 and MinSegLen = 10. Moreover, an average log2 ratio of ±0.25 was set as a cut-off value [25]. Among the detected CNVs, we selected those CNVs having a similar boundary with any CNV significant in the discovery association study. Additional file 1: Figure S2 shows the overall process of the replication study.

#### CNV validation of replication study samples

To verify whether an estimated CNV genotype using cohort samples is true or not, we carried out quantitative PCR (qPCR) using the TaqMan Copy number Assay (Life Technologies, Foster City, CA, USA) according to the manufacturer’s guidelines. A pre-designed TaqMan probe (Assay ID: Hs04965547_cn) was used to validate the existence of the CNV. All experiments were replicated three times to enhance the validation accuracy. The samples used for validation were randomly selected from each genotype (Additional file 1: Figure S1 (B)). Copy number genotype for each sample was calculated by Copy caller v2.0 (Applied Biosystems, Calif., USA) using the manufacturer’s guideline.

## Results

### Evaluation with simulated data

#### Clustering

With the simulated data we evaluated the accuracy of estimating *M* and the estimated copy numbers for each individual when the true *M* was assumed to be known. The results from the proposed method were compared with CNVtools [10]. The probe intensity measurements were generated under the three different scenarios: BSC, MSC and WSC. For each individual, we calculated from the probe intensity measurements the mean, the first PC score and the fewest PC scores that explain more than 90% of the variation; they are denoted by mean, PC1 and PC.9 respectively. In addition to the original probe intensity measurements (RAW), we used the mean, PC1 and PC.9, for the proposed method and the results were compared with CNVtools. For CNVtools, the mean, PC1 and the one-dimensional canonical correlation transformed vector of the probe intensity measurements were used.

Additional file 1: Tables S3 and S4 show the accuracy of the estimated value of *M* from 1000 replicates using PedCNV and CNVtools. The proposed method using PC1 was always the most accurate, followed by the proposed method using PC.9. The results from the proposed method performed better than CNVtools. CNVtools had a tendency to choose a larger number of clusters, and the results were rarely consistent, even when the clusters were well separated. CNVtools selects the number of clusters using a Bayesian information criterion [10], while the proposed method selects it with a silhouette score, which appears to be a better choice. In Additional file 1: Table S5, *M* was set to be the true value 3 for all methods, and the relative proportions of individuals for whom the estimated copy number was consistent with the true copy number were calculated from 1000 replicates under the null and alternative hypotheses. Additional file 1: Table S5 shows that the proposed method based on PC1 was the most accurate, followed by the proposed method based on PC.9. Therefore we conclude that basing our method on PC1 may be a reasonable choice.

#### Association analysis

**Fig. 1:**
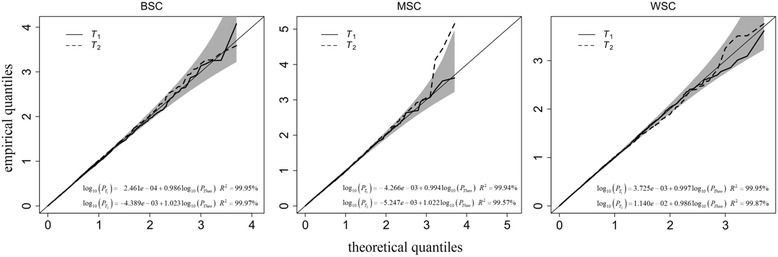
The QQ plots without genome control for *T*_*1*_ and *T*_*2*_ from simulated data. The empirical *p*-values adjusted by genomic control for the proposed methods were calculated under the null hypothesis with 5000 replicates under BSC, MSC and WSC, and their QQ plots are shown

In order to evaluate the proposed statistics *T*_1_ and *T*_2_, we simulated the probe intensity measures for BSC, MSC and WSC, and phenotypes were generated under the null and alternative hypotheses. Seven probe intensity measurements were generated, so that *T*_2_ followed the chi-square distribution with seven degrees of freedom under the null hypothesis. For the statistical validity of the proposed methods, empirical type-1 error estimates at the various significance levels were calculated from 5000 replicates; Table 2 shows that for our methods the nominal significance levels were always preserved under BSC, MSC and WSC. The quantile quantile (QQ) plots in Fig. 1 also indicate the validity of *T*_1_ and *T*_2_.

**Table 2:** Empirical type 1 error estimates (*M* = 3)

		Significance Level			
		.005	.05	.1	.2
BSC	T1	0.0060 ± 0.0021	0.0504 ± 0.0061	0.1018 ± 0.0084	0.2082 ± 0.0113
	T2	0.0056 ± 0.0021	0.0550 ± 0.0063	0.1008 ± 0.0086	0.2072 ± 0.0112
MSC	T1	0.0048 ± 0.0019	0.0486 ± 0.0060	0.1006 ± 0.0083	0.2104 ± 0.0113
	T2	0.0048 ± 0.0019	0.0472 ± 0.0059	0.0956 ± 0.0082	0.1884 ± 0.0108
WSC	T1	0.0056 ± 0.0021	0.0516 ± 0.0061	0.0962 ± 0.0082	0.2006 ± 0.0111
	T2	0.0048 ± 0.0019	0.0498 ± 0.0060	0.0968 ± 0.0082	0.1922 ± 0.0109

The 95% confidence intervals of empirical type I error estimates for the proposed methods were calculated from 5000 replicates at four significance levels under BSC, MSC and WSC, when there are three copy number clusters

To evaluate statistical efficiency, empirical power estimates for *T*_1_ and *T*_2_ were calculated from 2000 replicates under the alternative hypothesis, and compared with the FBAT statistic which directly utilizes the intensity measurement [11]. We considered various choices of *β*, and the probe intensity measurements for BSC, MSC and WSC were generated. Table 3 shows that the proposed statistics *T*_1_ and *T*_2_ performed better than FBAT, and *T*_1_ was always more powerful than *T*_2_ under all scenarios. The power loss of *T*_2_ compared to *T*_1_ is the largest for BSC and we can conclude that the statistical power of *T*_2_ is more affected by proportions of noise in the probe intensity measurement. We calculated empirical type 1 error and power estimates when *M* = 6 in Additional file 1: Tables S6 and S7, and the same patterns as *M* = 3 are observed. Moreover, we compared *T*_1_ with the statistic with the most probable copy number, and found that *T*_1_ is more powerful to estimate the parameters, especially under the BSC scenario (Additional file 1: Tables S8 and S9). Therefore, we conclude that *T*_1_ should be selected for a CNV association analysis.

**Table 3:** Empirical power estimates (*M* = 3)

Significance Level			*β*					
			.1	.2	.3	.4	.5	.6
.001	BSC	T1	0.0135	0.1390	0.4830	0.8410	0.9830	0.9985
		T2	0.0065	0.0510	0.2370	0.5745	0.8860	0.9795
		FBAT	5e-4	0.0060	0.0340	0.1300	0.3570	0.6005
	MSC	T1	0.0160	0.1570	0.5530	0.8740	0.9885	1.0000
		T2	0.0085	0.0685	0.3200	0.6900	0.9290	0.9945
		FBAT	0.0000	0.0100	0.0695	0.2505	0.5575	0.8385
	WSC	T1	0.0195	0.1615	0.5375	0.8935	0.9910	0.9980
		T2	0.0075	0.0815	0.3300	0.7240	0.9545	0.9970
		FBAT	0.0010	0.0115	0.0915	0.3240	0.6460	0.8955
.01	BSC	T1	0.0710	0.3585	0.7510	0.9605	0.9990	1.0000
		T2	0.0265	0.1780	0.4630	0.7900	0.9685	0.9950
		FBAT	0.0155	0.0455	0.1540	0.3775	0.6620	0.8430
	MSC	T1	0.0725	0.3805	0.8070	0.9690	0.9990	1.0000
		T2	0.0340	0.2100	0.5640	0.8660	0.9790	0.9980
		FBAT	0.0150	0.0550	0.2400	0.5385	0.8155	0.9645
	WSC	T1	0.0800	0.3795	0.7925	0.9740	0.9985	1.0000
		T2	0.0370	0.2115	0.5730	0.8855	0.9920	0.9995
		FBAT	0.0175	0.0710	0.2740	0.6350	0.8775	0.9740
.05	BSC	T1	0.1905	0.5930	0.9075	0.9920	1.0000	1.0000
		T2	0.0975	0.3505	0.6880	0.9080	0.9910	0.9990
		FBAT	0.0575	0.9610	0.3660	0.6310	0.8555	0.9525
	MSC	T1	0.2050	0.6190	0.9295	0.9900	1.0000	1.0000
		T2	0.1110	0.3915	0.7650	0.9495	0.9970	1.0000
		FBAT	0.0725	0.2080	0.4990	0.7585	0.9305	0.993
	WSC	T1	0.2095	0.6145	0.9260	0.9950	0.9995	1.0000
		T2	0.1255	0.3995	0.7650	0.9530	0.9990	1.0000
		FBAT	0.0790	0.2240	0.5460	0.8415	0.9705	0.9960

The empirical power for the proposed methods have been estimated at various significance levels based on 2000 replicates for different values of *β* under BSC, MSC and WSC, when there are three copy number clusters. The score test using the inferred CNVs is denoted by *T*_1_. The score test using the intensity measurements is denoted by *T*_2_. For comparison, we also calculated the power using FBAT

### Results of real data analysis

#### CNV association

**Fig. 2:**
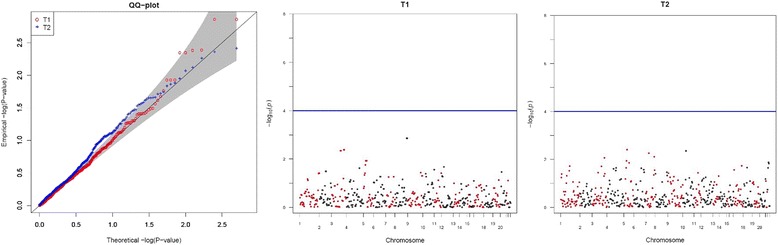
The QQ plot and Manhattan plots for *T*_*1*_ and *T*_*2*_ from analysis of the family data

500 well-separated multi-class CNVs were chosen for an association study. The 0.05 genome-wide significance level by Bonferroni correction for 500 CNVs is 10^−4^ and association analyses of Hct were conducted with the proposed methods. Figure 2 shows QQ and Manhattan plots for the statistics *T*_1_ and *T*_2_. We listed the most significant results of *T*_1_ and *T*_2_ respectively in Table 4. There is no genome-wide significant CNV and this is partially attributable to the insufficient sample size. In our analyses, 521 subjects are utilized, and if the effect size is 0.206 and sigma is 0.957, 1563 subjects are required to achieve 0.8 power at the 10^−4^ significant level. The difference between *T*_1_ and *T*_2_ may be attributable to the low accuracy of the clustering, because the performance of *T*_1_ depends on the accuracy of the clustering. However, *T*_2_ models the relationship between intensity and phenotypes without estimating copy numbers; but there is also the possibility of poor fit, including nonnormality.

**Table 4:** The most significant results of *T*_*1*_ and *T*_*2*_ from analyzing the family data

Chr	Position	0/1/2	*T*_1_	*T*_2_
8	94141469–94142527	42/226/253	1.38e-03	4.67e-02
5	147534018–147534337	119/265/137	1.19e-02	3.92e-03

#### CNV validation

Among 500 multi-class CNVs, the CNV region (chr7:81279592–81280418) was randomly selected for evaluation of CNV genotype estimation. In total, 41 subjects were selected from each CNV cluster and a PCR experiment was conducted for them. Among these samples, 38 subjects (92.7%) had the same copy numbers as the estimates from the proposed methods (Additional file 1: Figures S3 and S4).

## Discussion

Even though CNV has been expected to be an important genetic factor for many diseases, CNV association analysis has often been limited because of uncertainty of the copy number, and several statistical methods [8, 27] have been proposed to handle this uncertainty. However, even though some of the existing methods are relatively accurate, the estimated copy numbers are not accurate in some situations, which might cause a power loss for CNV association analysis. In this report, we propose new statistical methods for CNV association analysis with family-based samples. With extensive simulations, we showed that the proposed methods perform much better than the existing approaches. The proposed method was implemented in the R package, pedCNV and the main function in our R package was implemented with C++. We found that association analyses of 300 trios were completed within one minute using an Intel (R) Xeon (R) E5-2620 0 CPU at 2.00GHz, with a single node and 80 gigabyte memory.

Furthermore, the proposed method is flexible and can be extended to various scenarios. First, the proposed methods consist of *T*_1_ and *T*_2_. The former is based on the estimated copy number and the latter is on the probe intensity measurements. Our simulation studies show that the most efficient statistic is always the statistic with the expected copy numbers. However, if the accuracy of the estimated copy numbers is not clear and there is a systematic bias, the statistical power of *T*_1_ can be substantially affected, and some modification can be made to the proposed methods. For instance, the minimum of the *p*-values for *T*_1_ and *T*_2_ could be considered as a test statistic and permutation-based *p*-values could be calculated. Alternatively, the posterior probabilities for each copy number estimated from the E step in *T*_1_ can be utilized as classified copy numbers. These modifications are computationally feasible and may provide less sensitive results compared to *T*_1_ and *T*_2_. Second, the presence of population substructure has been known to be a factor that leads to violation of the assumptions underlying statistical association analysis. In our real data analysis, the genomic control approach [28] was adopted, but the linear mixed model is known to be the most efficient if the polygenic effects are substantial [29]. The correlations between individuals can be estimated with large-scale genetic data such as genome-wide SNPs, and this can be incorporated into the phenotype model in the proposed method. Third, the proposed methods can be simply extended to the sequencing data with a minor modification even though it only applied to aCGH data in this report. This will be investigated in our future work.

However, in spite of the practical advantage of the proposed methods, there exist some limitations, and further investigation is necessary. First, the incorporation of Mendelian transmission into the signal model induces a substantial computational burden for large families. In our PedCNV package, Mendelian transmission for a signal model is considered, but only for nuclear families. We found with simulation studies that the drop of accuracy is not substantial when Mendelian transmission is not considered, but its effect can be substantial if only a few large families are available. A peeling algorithm [30] has been developed that minimizes the computation of likelihoods for large families and it will be implemented in the PedCNV package. Second, the proposed method assumes that there is no de novo mutation and recombination. In such cases, the statistic *T*_2_ may be a better choice. Third, it has been observed that the bias in CNV calls can be different between parents and offspring, and our first statistic, *T*_1_, can suffer from this differential bias. Our simulation studies do not examine any such violation of statistical assumptions, but its effect on *T*_1_ could be substantial in CNV association analysis with large families. Third, copy numbers for each individual were identified by calculating the expectation of copy numbers using the posterior probability and the expected copy numbers were utilized as λ in *T*_1_. Although this maximum likelihood approach for classification can yield inconsistent estimators of parameters [31, 32], the simulation studies show that the accuracy of this method is higher. Thus we continued adopting this method in spite of its deficiencies.

In recent decades various types of genetic data have been used to detect the genetic factors underlying many diseases and many disease susceptibility loci have been found. Even though CNVs have been expected to be an important genetic factor, the findings of CNV association analysis have been limited and the proposed methods may bridge this gap by alleviating the issue of copy number uncertainty.

## Conclusion

PedCNV presents a computationally efficient R package that provides two statistics for family-based CNV association analysis: first, the copy number is estimated by maximum likelihood and association of the estimated copy number with the phenotype is tested; second, the observed probe intensity measurements is directly used to detect association of CNV with the phenotypes of. The simulation studies showed that the proposed methods outperform other existing approaches. In particular, we found that statistical analysis with the expected copy number is more powerful than the statistic with the probe intensity measurements regardless of the accuracy of the estimation of copy numbers.

## Additional files

Additional file 1: A complete report for all experiments performed for this work. Text 1. Rao’s score test statistic with the expected copy number. Text 2. Rao’s score test statistic with the probe intensity measurements. **Figure S1.** Results of the clustering analysis with family samples (A) and cohort samples (B). **Figure S2.** Schematic representation of the strategy for the CNV analysis. **Figure S3.** Validation results. **Figure S4.** Validation results of replication samples by TaqMan qPCR experiment. **Table S1.** Specification of parameters for the signal model used in the simulation studies. **Table S2.** Primer information for CNV validation. **Table S3.** Accuracy of copy number clusters identified with PedCNV. **Table S4.** Accuracy of copy number clusters identified with CNVtools. **Table S5.** Accuracy of copy number estimated with silhouette score. **Table S6.** Empirical type 1 error estimates when *M* = 6. **Table S7.** Empirical power estimates when *M* = 6. **Table S8.** Empirical power estimates of *T*_1_ and *T*_1_^*^ which use the expected copy number and the most probable copy number respectively. **Table S9.** Estimated parameters of *T*_1_ and *T*_1_^*^ which use the expected copy number and the most probable copy number respectively. (PDF 814 kb)

Additional file 2: Simulated data with 2000 replicates for different values of *β* under WSC, when there are three copy number clusters. (ZIP 138685 kb)

## Abbreviations

aCGH: array comparative genomic hybridization
BSC: Badly separated clusters
CNV: Copy number variation
GADA: Genome Alteration Detection Analysis
Hct: Hematocrit
KoGES: Korean Genome Epidemiology Study
LCL: Lymphoblastoid cell line
MSC: Moderately separated clusters
PC: Principle component
qPCR: quantitative PCR
QQ plot: Quantile quantile plot
REML: Restricted maximum likelihood
WSC: Well separated clusters
