# Supplementary material for: On the association analysis of CNV data: a fast and robust family-based association method
Source: BMC Bioinformatics. 2017 Apr 18;18:217. doi: 10.1186/s12859-017-1622-z (PMC5395793; doi:10.1186/s12859-017-1622-z)
Supplement: Supplementary file 1 — A complete report for all experiments performed for this work. Text 1. Rao’s score test statistic with the expected copy number. Text 2. Rao’s score test statistic with the probe intensity measurements. Figure S1. Results of the clustering analysis with family samples (A) and cohort samples (B). Figure S2. Schematic representation of the strategy for the CNV analysis. Figure S3. Validation results. Figure S4. Validation results of replication samples by TaqMan qPCR experiment. Table S1. Specification of parameters for the signal model used in the simulation studies. Table S2. Primer information for CNV validation. Table S3. Accuracy of copy number clusters identified with PedCNV. Table S4. Accuracy of copy number clusters identified with CNVtools. Table S5. Accuracy of copy number estimated with silhouette score. Table S6. Empirical type 1 error estimates when M = 6. Table S7. Empirical power estimates when M = 6. Table S8. Empirical power estimates of T 1 and T 1 * which use the expected copy number and the most probable copy number respectively. Table S9. Estimated parameters of T 1 and T 1 * which use the expected copy number and the most probable copy number respectively. (PDF 814 kb) [file 12859_2017_1622_MOESM1_ESM.pdf]

Web-based Supplementary Materials for "On the Association Analysis of CNV Data: a Fast and Robust Family-based Association Method" by Meiling Liu, Sanghoon Moon, Longfei Wang, Sulgi Kim, Yeon-Jung Kim, Mi Yeong Hwang, Young Jin Kim, Robert C Elston, Bong-Jo Kim, and Sungho Won

Supplementary Text 1 Rao's score test statistic with the expected copy number

We let $\mathbf{V} = \sigma_g^2\mathbf{\Phi} + \sigma_\varepsilon^2\mathbf{I}_N$, $\mathbf{e} = \mathbf{Y} - \mathbf{Za} - \boldsymbol{\lambda}\beta$ and let ξ denotes all the parameters expect for β and $\boldsymbol{\alpha}$. If we assume that the copy number for each individual is known, the conditional log density function of Y is

$$l(\beta, \xi; \boldsymbol{Y}|\boldsymbol{\lambda}) = -\frac{1}{2}log|\boldsymbol{V}| - \frac{1}{2}\boldsymbol{e}^T\boldsymbol{V}^{-1}\boldsymbol{e}.$$

The score for the proposed likelihood is thus

$$S_1(\beta) = \frac{\partial l}{\partial \beta} = \mathbf{e}^T\mathbf{V}^{-1}\boldsymbol{\lambda}.$$

Instead of the expected Fisher information matrix, the hybrid method has been used – that is, the nonzero elements of the Fisher information are replaced by the observed information(Kent, 1982). Then, under the null hypothesis, the information is

$$i_f = \begin{bmatrix} i_{f11} & i_{f12} \\ i_{f21} & i_{f22} \end{bmatrix}, \quad i_{f11} = \boldsymbol{\lambda}^T\mathbf{V}^{-1}\boldsymbol{\lambda}, \quad i_{f12} = ((\mathbf{Z}^T\mathbf{V}^{-1}\boldsymbol{\lambda})^T \quad \mathbf{0} \quad \mathbf{0}) = i_{f21}^t, \quad i_{f22} = \begin{bmatrix} \mathbf{Z}^T\mathbf{V}^{-1}\mathbf{Z} & \mathbf{0} \\ \mathbf{0} & \mathbf{A} \end{bmatrix},$$

where

$$\mathbf{A} = \begin{bmatrix} \frac{1}{2}\mathrm{tr}(\mathbf{V}^{-1}\mathbf{\Phi}\mathbf{V}^{-1}\mathbf{\Phi}) - \mathbf{e}^T\mathbf{V}^{-1}\mathbf{\Phi}\mathbf{V}^{-1}\mathbf{\Phi}\mathbf{V}^{-1}\mathbf{e} & \frac{1}{2}\mathrm{tr}(\mathbf{V}^{-1}\mathbf{V}^{-1}\mathbf{\Phi}) - \mathbf{e}^T\mathbf{V}^{-1}\mathbf{\Phi}\mathbf{V}^{-1}\mathbf{V}^{-1}\mathbf{e} \\ \frac{1}{2}\mathrm{tr}(\mathbf{V}^{-1}\mathbf{V}^{-1}\mathbf{\Phi}) - \mathbf{e}^T\mathbf{V}^{-1}\mathbf{\Phi}\mathbf{V}^{-1}\mathbf{V}^{-1}\mathbf{e} & \frac{1}{2}\mathrm{tr}(\mathbf{V}^{-1}\mathbf{V}^{-1}) - \mathbf{e}^T\mathbf{V}^{-1}\mathbf{V}^{-1}\mathbf{V}^{-1}\mathbf{e} \end{bmatrix},$$

Because $i_{f12}i_{f22}^{-1}i_{f21}$ is

$$[(\mathbf{Z}^T\mathbf{V}^{-1}\boldsymbol{\lambda})^T \quad \mathbf{0} \quad \mathbf{0}] \begin{bmatrix} (\mathbf{Z}^T\mathbf{V}^{-1}\mathbf{Z})^{-1} & \mathbf{0} \\ \mathbf{0} & \mathbf{A}^{-1} \end{bmatrix} \begin{bmatrix} \mathbf{Z}^T\mathbf{V}^{-1}\boldsymbol{\lambda} \\ \mathbf{0} \\ \mathbf{0} \end{bmatrix} = (\mathbf{Z}^T\mathbf{V}^{-1}\boldsymbol{\lambda})^T(\mathbf{Z}^T\mathbf{V}^{-1}\mathbf{Z})^{-1}(\mathbf{Z}^T\mathbf{V}^{-1}\boldsymbol{\lambda}),$$

the score test statistic T_1 becomes

$$T_1 = ((\mathbf{Y} - \mathbf{Z}\boldsymbol{\alpha})^T\mathbf{V}^{-1}\boldsymbol{\lambda})^T\left(\boldsymbol{\lambda}^T\mathbf{V}^{-1}\boldsymbol{\lambda} - (\mathbf{Z}^T\mathbf{V}^{-1}\boldsymbol{\lambda})^T(\mathbf{Z}^T\mathbf{V}^{-1}\mathbf{Z})^{-1}(\mathbf{Z}^T\mathbf{V}^{-1}\boldsymbol{\lambda})\right)^{-1}((\mathbf{Y} - \mathbf{Z}\boldsymbol{\alpha})^T\mathbf{V}^{-1}\boldsymbol{\lambda}),$$

and T_1 follows the chi-square distribution with a single degree of freedom under H_0.

Supplementary Text 2 Rao's score test statistic with the probe intensity measurements

If the unobserved copy number vector λ is given, the score for β is

$$\mathbf{e}^T\mathbf{V}^{-1}\boldsymbol{\lambda}.$$

The copy number is unknown and is replaced by the expected score for β as follows:

$$\sum\nolimits_{\lambda}\mathbf{e}^T\mathbf{V}^{-1}\boldsymbol{\lambda}P(\boldsymbol{\lambda}) = \mathbf{e}^T\mathbf{V}^{-1}E(\boldsymbol{\lambda}).$$

We assume that $E(\lambda|\mathrm{X})$ has a linear relationship with the probe intensity measurements and, if we let $\mathbf{S}_1 = \mathbf{Z}(\mathbf{Z}^T\mathbf{V}^{-1}\mathbf{Z})^{-1}\mathbf{Z}^T\mathbf{V}^{-1}$, our score under the null hypothesis is equivalent to

$$\mathbf{Y}^T(\mathbf{I}_N - \mathbf{S}_1)^T\mathbf{V}^{-1}\mathbf{X}\gamma.$$

Let $\mathbf{S}_2 = \mathbf{1}_N(\mathbf{1}_N^T\boldsymbol{\Phi}^{-1}\mathbf{1}_N)^{-1}\mathbf{1}_N^T\boldsymbol{\Phi}^{-1}$, and define

$$u_k = \mathbf{Y}^T(\mathbf{I}_N - \mathbf{S}_1)^T\mathbf{V}^{-1}(\mathbf{I}_N - \mathbf{S}_2)\mathbf{X}^k.$$

We let $\mathbf{X}^k = \left(x_{11k}, \ldots, x_{nn_nk}\right)^T$ and we assume that $\mathrm{cov}\left(\mathbf{X}^k, \mathbf{X}^{k'}\right) = \psi_{kk'}\boldsymbol{\Psi}$. The Mendelian transmission of copy numbers make $\boldsymbol{\Psi}$ similar to $\boldsymbol{\Phi}$ but some deviation is expected because of the presence of measurement error. Therefore, the empirical estimator for $\boldsymbol{\Psi}$ is utilized. Denoting the covariance between x_{ijk} and $x_{ijk'}$ by ψ'_{kk} and letting

$$\boldsymbol{\Psi} = \begin{bmatrix} \psi_{11} & \cdots & \psi_{K1} \\ \vdots & \ddots & \vdots \\ \psi_{1K} & \cdots & \psi_{KK} \end{bmatrix},$$

we have

$$E\left[\mathbf{X}^{kT}(\mathbf{I}_N - \mathbf{S}_2)^T\boldsymbol{\Phi}^{-1}(\mathbf{I}_N - \mathbf{S}_2)\mathbf{X}^{k'}\right] = \psi_{kk'}tr\{(\mathbf{I}_N - \mathbf{S}_2)\boldsymbol{\Psi}\boldsymbol{\Phi}^{-1}\},$$

and $\boldsymbol{\Psi}$ is estimated by

$$\widehat{\boldsymbol{\Psi}} = \frac{1}{tr\{(\mathbf{I}_N - \mathbf{S}_2)\boldsymbol{\Psi}\boldsymbol{\Phi}^{-1}\}}\mathbf{X}^T(\mathbf{I}_N - \mathbf{S}_2)^T\boldsymbol{\Phi}^{-1}(\mathbf{I}_N - \mathbf{S}_2)\mathbf{X}.$$

It should be noted that if $\boldsymbol{\Psi}$ is equal to $\boldsymbol{\Phi}$,

$$\widehat{\boldsymbol{\Psi}} = \frac{1}{N-1}\mathbf{X}^T(\mathbf{I}_N - \mathbf{S}_2)^T\boldsymbol{\Phi}^{-1}(\mathbf{I}_N - \mathbf{S}_2)\mathbf{X}.$$

We can simply show that

$$\mathrm{var}(\mathbf{V}^{-1}(\mathbf{I}_N - \mathbf{S}_1)\mathbf{Y}) = \mathbf{V}^{-1}(\mathbf{I}_N - \mathbf{S}_1)\mathbf{V}(\mathbf{I}_N - \mathbf{S}_1)^T\mathbf{V}^{-1} = \mathbf{V}^{-1}(\mathbf{I}_N - \mathbf{S}_1),$$

and

$$\mathrm{cov}\left((\mathbf{I}_N - \mathbf{S}_2)\mathbf{X}^k, (\mathbf{I}_N - \mathbf{S}_2)\mathbf{X}^{k'}\right) = \psi_{kk'}(\mathbf{I}_N - \mathbf{S}_2)\boldsymbol{\Psi}(\mathbf{I}_N - \mathbf{S}_2)^T.$$

In addition $E(\mathbf{V}^{-1}(\mathbf{I}_N - \mathbf{S}_1)\mathbf{Y}) = 0, and\ E((\mathbf{I}_N - \mathbf{S}_2)\mathbf{X}^k) = 0$, and thus under the null hypothesis $\mathrm{cov}(u_k, u'_k)$ is

$$E\left[\{\mathbf{Y}^T(\mathbf{I}_N - \mathbf{S}_1)\mathbf{V}^{-1}(\mathbf{I}_N - \mathbf{S}_2)\mathbf{X}^k\}\{\mathbf{Y}^T(\mathbf{I}_N - \mathbf{S}_1)\mathbf{V}^{-1}(\mathbf{I}_N - \mathbf{S}_2)\mathbf{X}^{k'}\}^T\right]$$

$$= \mathrm{tr}\left[\mathrm{cov}\left((\mathbf{I}_N - \mathbf{S}_2)\mathbf{X}^k, (\mathbf{I}_N - \mathbf{S}_2)\mathbf{X}^{k'}\right)\mathrm{var}(\mathbf{V}^{-1}(\mathbf{I}_N - \mathbf{S}_1)\mathbf{Y})\right]$$

$$= \psi_{kk'}\mathrm{tr}[(\mathbf{I}_N - \mathbf{S}_2)\boldsymbol{\Psi}(\mathbf{I}_N - \mathbf{S}_2)^T\mathbf{V}^{-1}(\mathbf{I}_N - \mathbf{S}_1)].$$

If we let $\mathbf{u} = (u_1, \ldots, u_K)^T$, the variance-covariance matrix of u is

$$\mathbf{v} = \{\mathrm{tr}[(\mathbf{I}_N - \mathbf{S}_2)\boldsymbol{\Psi}(\mathbf{I}_N - \mathbf{S}_2)^T\mathbf{V}^{-1}(\mathbf{I}_N - \mathbf{S}_1)]\}\widehat{\boldsymbol{\Psi}}.$$

Maximizing the function $\gamma^T\mathbf{u}(\gamma^T\mathbf{v}\gamma)^{-1}\mathbf{u}^T\gamma$ with respect to γ yields the test statistic

$$T_2 = \mathbf{u}^T\mathbf{v}^{-1}\mathbf{u},$$

and T_2 follows the chi-square distribution with degrees of freedom equal to the rank of $\boldsymbol{v}$.

Supplementary Figure 1. Results of the clustering analysis with family samples (A) and cohort samples (B).

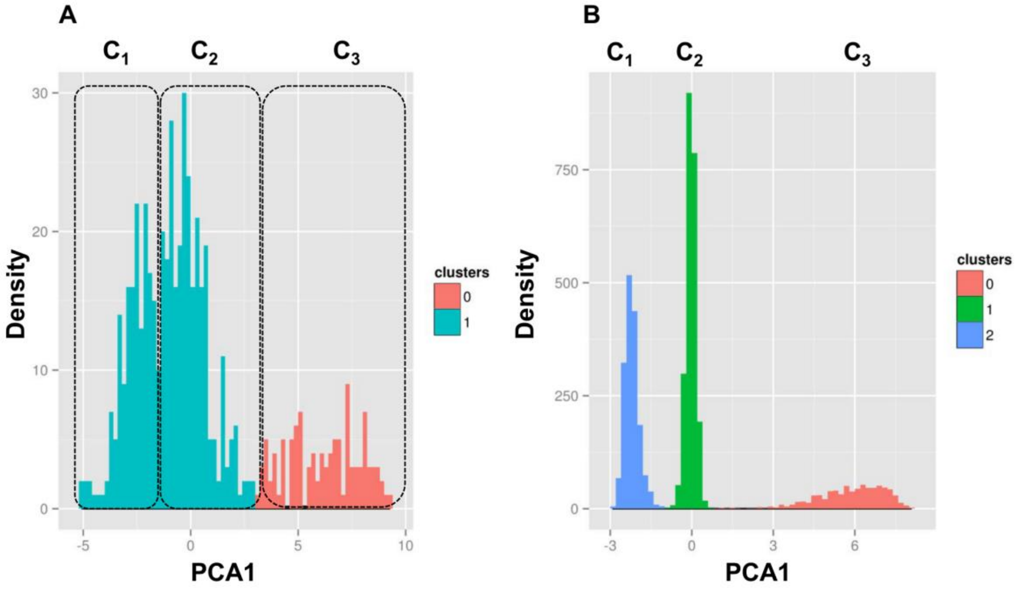

(A) Histogram depicts clustering result using log2 ratio values based on signal intensity of the reference and each sample. PedCNV calculated two copy-number classes from the family samples, but we estimated that this region is composed of three copy-number classes denoted as C1, C2, and C3. This discrepancy of copy-number genotype might be caused by the difference of sample size between the two studies. Moreover, because the T2 statistic of PedCNV is robust to badly separated clusters, this bias of CNV call cannot affect association results. However, to evaluate the exact CNV genotype, we chose samples from C1, C2, and C3 to carry out validation experiments. (B) Histogram represents clustering result from the community-based cohort. Different from to the clustering result from the analysis of family samples, there are three well-separated copy-number classes.

Supplementary Figure 2. Schematic representation of the strategy for the CNV analysis.

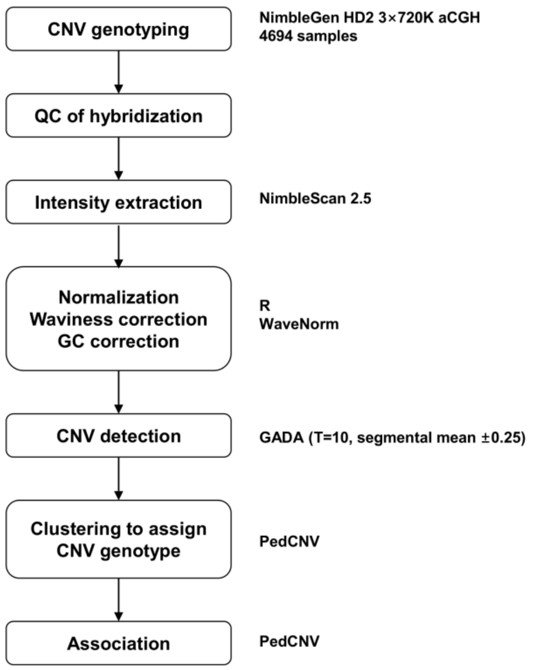

Supplementary Figure 3. Validation results.

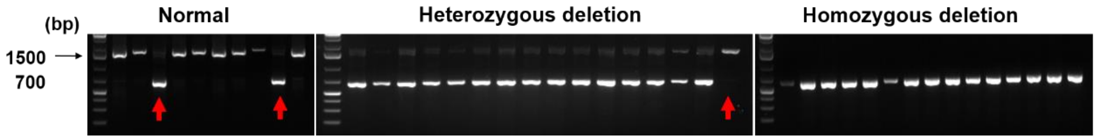

Validation results by PCR experiment. The PCR experiment was conducted to evaluate concordance between genotypes and copy-number estimates within each cluster (normal, heterozygous deletion and homozygous deletion). The PCR product size of the normal allele was 1519bp, whereas that of deleted allele was 690bp. Validation results show complete concordance with estimated genotypes except for 3 samples (red arrows).

Supplementary Figure 4. Validation results of replication samples by TaqMan qPCR experiment.

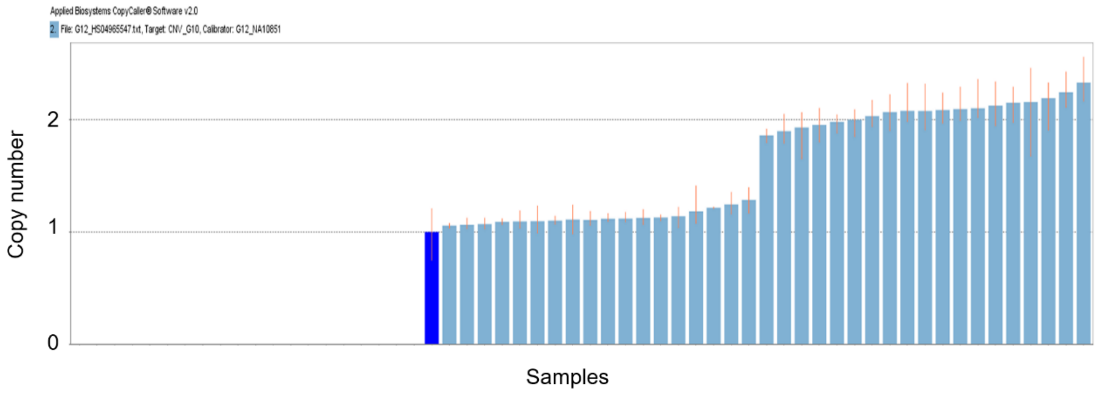

Supplementary Table 1. Specification of parameters for the signal model used in the simulation studies.

	s_1, s_2, s_3	s_4, s_5, s_6, s_7	z
BSC	1.5	0.15	0.2
MSC	2.5	0.25	
WSC	4.0	0.40	

The parameters are shown for badly separated clusters (BSC), moderately separated clusters (MSC) and well separated clusters (WSC).

Supplementary Table 2. Primer information for CNV validation

Sequence	Start (hg19)	End (hg19)	Primer	Size (bp)
TTCTGTTGTGGACTGGCATG	81441341	81441360	Forward	1519
GTGGTGAGTTACGAGCATAG	81442860	81442879	Reverse	

Genomic locations for the designed primers based on human genome assembly hg18 were converted to those based on hg19 by liftOver of the UCSC genome browser.

Supplementary Table 3. Accuracy of copy number clusters identified with PedCNV.

MEAN			PC1			PC.9			RAW		
BSC	MSC	WSC	BSC	MSC	WSC	BSC	MSC	WSC	BSC	MSC	WSC
0.013	0.132	0.310	0.998	1	1	0	0.891	1	0	0.002	0.007

Among 1,000 replicates, the proportions of replicates correctly identified the number of clusters with PedCNV were calculated under BSC, MSC and WSC.

Supplementary Table 4. Accuracy of copy number clusters identified with CNVtools.

Mean			PC1			LDF		
BSC	MSC	WSC	BSC	MSC	WSC	BSC	MSC	WSC
0	0	0.01	0	0	0	0	0	0

Among 1,000 replicates, the proportions of replicates correctly identified the number of clusters with CNVtools were calculated under BSC, MSC and WSC.

Supplementary Table 5. Accuracy of copy number estimated with silhouette score.

	MEAN			PC1			PC.9			RAW		
	BSC	MSC	WSC	BSC	MSC	WSC	BSC	MSC	WSC	BSC	MSC	WSC
β=0	0.840	0.962	0.996	0.947	0.995	0.999	0.519	0.995	0.999	0.515	0.996	0.999
β=0.1	0.812	0.961	0.996	0.947	0.995	0.999	0.490	0.995	0.999	0.487	0.995	0.999

The copy number *M* was estimated with silhouette score and the proportions of replicates which correctly identified *M* among 1000 replicates were calculated under BSC, MSC and WSC.

Supplementary Table 6. Empirical type 1 error estimates when *M* = 6.

		Significance Level			
		.005	.05	.1	.2
BSC	T_1	0.0040±0.0028	0.0475±0.0093	0.0935±0.0128	0.1940±0.0173
	T_2	0.0045±0.0029	0.0480±0.0094	0.0945±0.0128	0.2015±0.0176
MSC	T_1	0.0040±0.0028	0.0445±0.0090	0.1015±0.0132	0.2015±0.0176
	T_2	0.0030±0.0024	0.0495±0.0095	0.0910±0.0126	0.1800±0.0168
WSC	T_1	0.0060±0.0034	0.0510±0.0094	0.1090±0.0137	0.2140±0.0180
	T_2	0.0030±0.0024	0.0485±0.0094	0.1000±0.0132	0.1995±0.0175

We assume *M*=6, and empirical type I error estimates and their 95% confidence intervals for the proposed methods were calculated from 2,000 replicates at four significance levels under BSC, MSC and WSC.

Supplementary Table 7. Empirical power estimates when M = 6.

Significance Level			β					
			.1	.2	.3	.4	.5	.6
.001	BSC	T_1	0.048	0.543	0.975	1.000	1.000	1.000
		T_2	0.010	0.170	0.715	0.987	0.999	1.000
	MSC	T_1	0.057	0.600	0.975	1.000	1.000	1.000
		T_2	0.011	0.218	0.802	0.999	1.000	1.000
	WSC	T_1	0.057	0.617	0.977	1.000	1.000	1.000
		T_2	0.009	0.234	0.817	0.994	1.000	1.000
.01	BSC	T_1	0.202	0.807	0.994	1.000	1.000	1.000
		T_2	0.048	0.420	0.913	0.997	1.000	1.000
	MSC	T_1	0.201	0.845	0.995	1.000	1.000	1.000
		T_2	0.061	0.486	0.937	1.000	1.000	1.000
	WSC	T_1	0.203	0.837	0.988	1.000	1.000	1.000
		T_2	0.066	0.488	0.945	1.000	1.000	1.000
.05	BSC	T_1	0.405	0.940	0.999	1.000	1.000	1.000
		T_2	0.176	0.675	0.966	1.000	1.000	1.000
	MSC	T_1	0.420	0.950	1.000	1.000	1.000	1.000
		T_2	0.192	0.733	0.985	1.000	1.000	1.000
	WSC	T_1	0.426	0.946	1.000	1.000	1.000	1.000
		T_2	0.205	0.721	0.987	1.000	1.000	1.000

We assumed M = 6, and the empirical power estimates for the proposed were calculated at various significance levels with 1,000 replicates for different values of β under BSC, MSC and WSC.

Supplementary Table 8. Empirical power estimates of T_1 and T_1^* which use the expected copy number and the most probable copy number respectively.

Significance Level			β .1	.2	.3	.4	.5	.6
.001	BSC	T_1	0.0135	0.1390	0.4830	0.8410	0.9830	0.9985
		$\boldsymbol{T_1^*}$	0.0110	0.1320	0.4660	0.8320	0.9840	0.9980
	MSC	T_1	0.0160	0.1570	0.5530	0.8740	0.9885	1.0000
		$\boldsymbol{T_1^*}$	0.0155	0.1545	0.5490	0.8735	0.9900	0.9990
	WSC	T_1	0.0195	0.1615	0.5375	0.8935	0.9910	0.9980
		$\boldsymbol{T_1^*}$	0.0175	0.1615	0.5315	0.8885	0.9900	0.9985
.01	BSC	T_1	0.0710	0.3585	0.7510	0.9605	0.9990	1.0000
		$\boldsymbol{T_1^*}$	0.0665	0.3485	0.7360	0.9540	0.9990	1.0000
	MSC	T_1	0.0725	0.3805	0.8070	0.9690	0.9990	1.0000
		$\boldsymbol{T_1^*}$	0.0725	0.3795	0.8060	0.9695	0.9995	1.0000
	WSC	T_1	0.0800	0.3795	0.7925	0.9740	0.9985	1.0000
		$\boldsymbol{T_1^*}$	0.0780	0.3750	0.7895	0.9755	0.9990	1.0000
.05	BSC	T_1	0.1905	0.5930	0.9075	0.9920	1.0000	1.0000
		$\boldsymbol{T_1^*}$	0.1815	0.5915	0.8985	0.9910	1.0000	1.0000
	MSC	T_1	0.2050	0.6190	0.9295	0.9900	1.0000	1.0000
		$\boldsymbol{T_1^*}$	0.2070	0.6195	0.9260	0.9910	1.0000	1.0000
	WSC	T_1	0.2095	0.6145	0.9260	0.9950	0.9995	1.0000
		$\boldsymbol{T_1^*}$	0.2070	0.6110	0.9265	0.9950	1.0000	1.0000

The empirical powers of T_1 and T_1^* which use the expected copy number and the most probable copy number respectively were estimated at various significance levels with 2,000 replicates for different values of β under BSC, MSC and WSC. The score test using the expected copy number is denoted by T_1.

Supplementary Table 9. Estimated parameters of T_1 and T_1^* which use the expected copy number and the most probable copy number respectively.

			β			
			.1	.2	.3	.4
BSC	beta	T_1	0.0995±0.0025	0.1996±0.0026	0.2981±0.0026	0.3953±0.0026
		T_1^*	0.0963±0.0025	0.1945±0.0025	0.2896±0.0025	0.3843±0.0025
	alpha0	T_1	0.0010±0.0018	0.0031±0.0018	0.0042±0.0018	0.0083±0.0018
		T_1^*	0.0025±0.0018	0.0060±0.0017	0.0086±0.0017	0.0143±0.0018
	alpha1	T_1	0.1000±0.0012	0.0986±0.0012	0.0990±0.0012	0.1008±0.0012
		T_1^*	0.0999±0.0012	0.0986±0.0012	0.0991±0.0012	0.1007±0.0012
MSC	beta	T_1	0.0982±0.0024	0.1987±0.0025	0.3005±0.0024	0.3947±0.0025
		T_1^*	0.0979±0.0024	0.1980±0.0025	0.2998±0.0025	0.3923±0.0025
	alpha0	T_1	-0.0004±0.0018	-0.0012±0.0018	-0.0008±0.0018	-0.0002±0.0018
		T_1^*	0.0000±0.0018	-0.0009±0.0018	-0.0003±0.0018	0.0004±0.0018
	alpha1	T_1	0.1013±0.0013	0.1002±0.0013	0.0989±0.0012	0.1012±0.0013
		T_1^*	0.1014±0.0013	0.1001±0.0013	0.0989±0.0013	0.1013±0.0013
WSC	beta	T_1	01005±0.0025	0.1988±0.0025	0.2975±0.0025	0.3953±0.0025
		T_1^*	0.1002±0.0024	0.1983±0.0025	0.2971±0.0025	0.3923±0.0025
	alpha0	T_1	0.0000±0.0018	-0.0012±0.0018	-0.0016±0.0018	-0.0018±0.0018
		T_1^*	-0.0001±0.0018	-0.0011±0.0018	-0.0013±0.0018	-0.0020±0.0018
	alpha1	T_1	0.0997±0.0012	0.0976±0.0012	0.1007±0.0012	0.1002±0.0013
		T_1^*	0.1000±0.0012	0.0976±0.0012	0.1006±0.0012	0.1001±0.0013

The estimated parameters of T_1 and T_1^* which use the expected copy number and the most probable copy number respectively were estimated at various significance levels with 2,000 replicates for different values of β under BSC, MSC and WSC. The score test using the expected copy number is denoted by T_1.
